# An antibody staining protocol variation for nematodes that adds heat-induced antigen retrieval (HIAR)

**DOI:** 10.17912/micropub.biology.000135

**Published:** 2019-08-29

**Authors:** Curtis Loer, Hanh Witte, Ralf J Sommer, Oliver Hobert

**Affiliations:** 1 Department of Biology, University of San Diego, CA, USA; 2 Max Planck Institute for Developmental Biology, Tübingen, Germany; 3 Department of Biological Sciences, HHMI, Columbia University, NY, USA [CL, while visiting scholar on sabbatical]

**Figure 1.**
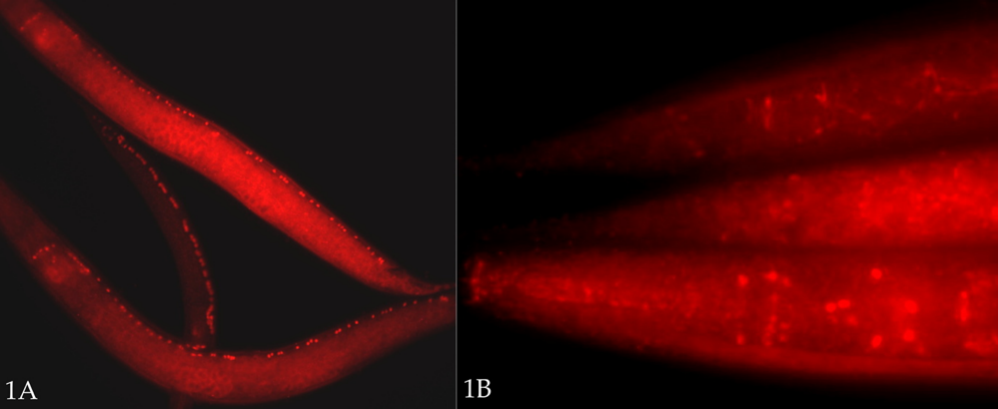
Figure 1: FLAG antibody-stained *P. pacificus* worms using the HIAR-added protocol, as described in the text. Details on generating CRISPR/Cas9-mediated FLAG-epitope tagged alleles, and precise sites and developmental time courses of nuclear expression will be reported elsewhere. (A) Three *Ppa-unc-3::2xFLAG* larval worms (RS3506) stained with mouse anti-FLAG monoclonal antibody plus Alexa-594-conjugated anti-mouse secondary. (B) Staining of *Ppa-unc-86::1xFLAG* strain (RS3509) with anti-FLAG – two tails (top), one head (bottom) of larve; antibodies as in A.

## Description

In the nematode satellite model organism *Pristionchus pacificus*, transgenics and CRISPR/Cas9 have been implemented in the last decade (Schlager *et al*., 2009; Cinkornpumin & Hong, 2011; Witte *et al*., 2015; Namai & Sugimoto, 2018). While CRISPR has been used to make mutants and to knock-in short sequences, sequence insertion to date is limited to ~150 base pairs. Therefore, following expression from native loci in *P. pacificus* currently depends on epitope-tagging the gene of interest via CRISPR and template-dependent repair, and then using immunocytochemistry with its maddening, nearly infinite possible variations in fixation, permeabilization, blocking, and antibody incubation protocols.

We used CRISPR/Cas9 to epitope-tag *P. pacificus* orthologs of two transcription factor genes known to function in neuronal specification in *C. elegans:* *unc-3* (Prasad *et al*., 1998; Kratsios *et al*., 2011) and *unc-86* (Finney and Ruvkun, 1990; Zhang *et al*., 2014). *Ppa-unc-3* was tagged at the C-terminus with 2x-FLAG; a single FLAG tag was added to the C-terminus of *Ppa-unc-86*. Using an ‘Improved Finney-Ruvkun’-style fixation, permeabilization and antibody incubation method (similar to Bettinger *et al*., 1996; Finney & Ruvkun, 1990; and ‘Peroxide tube fixation’ described in Duerr, 2006), we observed strong nuclear staining in neurons in four independent *Ppa-unc-3::2xFLAG* strains, although the staining was highly variable both within and between preps (and worm stages), and only a small subset of worms stained well. On the contrary, in three independent *Ppa-unc-86::1xFLAG* strains, we never saw any nuclear staining. After repeated attempts with some variations proved unsuccessful, we included a treatment long-known and widely-used with formalin-fixed and paraffin-embedded tissues in the field of pathology (Shi *et al*., 1990; reviewed by Yamashita, 2007), but apparently neglected in the worm field: heat-induced antigen retrieval (HIAR), also known as heat-induced epitope retrieval (HIER). In this method, after fixation, tissue is heated to boiling temperature (or higher, under pressure), typically for a short time (~60 min or less) using boiling water, microwave, autoclave, pressure cooker, etc. Likely the main mechanism of HIAR is breaking covalent bonds between proteins and other components formed during fixation, making antigens accessible by unmasking and changing their conformation, and perhaps also enhancing overall permeability of the tissue. HIAR is highly pH-dependent, working in low (<4) or high (>8) pH solutions, with high pH generally preferred for better tissue preservation (see review by Yamashita, 2007). In one study, HIAR was found to be essential for antibody staining against all 9 tested nuclear antigens, and 7 of 26 cytoplasmic and cell membrane antigens in ethanol-fixed human tissues (Denda *et al*., 2012).

We treated fixed *P. pacificus* worms in BO_3_ (borate) buffer [10 mM H_3_BO_3_, 10 mM NaOH, 1% Triton X-100, pH ~9] in an Eppendorf ‘Thermomixer’ block at 99C (maximal setting) for 15 or 30 min, prior to incubation/‘blocking’ in protein-containing ‘Antibody Buffer B’ solution (see below). In the case of *unc-3::2xFLAG* strains, both 15 and 30 minute heat treatments dramatically improved the staining – greatly reducing variability, with almost all animals staining moderately well to very well (Fig. 1A). For the *unc-86*:*:1xFLAG* strain, for the first time we saw nuclear staining in many worms (Fig. 1B). (The expression patterns of both *Ppa-unc-3::2xFLAG* and *Ppa-unc-86::1xFLAG* strains will be described elsewhere. It should also be noted that both a mouse anti-FLAG monoclonal and rabbit anti-FLAG polyclonal antibody recognized an epitope in wild type *P. pacificus* worms that appears to mark cell junctions, especially in the intestine. This does not typically interfere with identification of neuronal nuclei in the strains described.) One disadvantage to the HIAR treatment in our preparations is an increased variability and reduction – or loss – in DAPI staining, with high background. Normally, this fixation and permeabilization method (minus HIAR) yields uniformly excellent DAPI staining. With HIAR, in some cases, we observed the reduction or loss of staining to be selective: smaller, more compact nuclei such as those of sperm and neurons retained their DAPI staining better than others (e.g., hypodermal and intestinal nuclei). A shorter HIAR treatment (15 min) appears to preserve DAPI staining better. In a separate experiment, a 5 min HIAR treatment was insufficient to reveal more than a trace of *Ppa-unc-86::1xFLAG* staining compared to 15 and 30 min treatments, although DAPI staining was better preserved.

In conclusion, if antibody staining experiments with conventional protocols are not working well, we suggest adding HIAR to the end of a standard protocol prior to blocking and antibody incubation. As a potential caveat, we note it is possible that this works well only for antigens like the FLAG epitope, and/or for these nuclear antigens, and not with others. We have not yet tried HIAR with other antigens or other fixation methods.

## Methods

[derived from a Hobert lab protocol entitled ‘Improved Finney-Runkvun’; very similar to the protocol described in Bettinger *et al*., 1996]

1. Grow sufficient worms (synchronized, if desired) on 60 mm or 100 mm plates. A densely populated 60 mm plate has sufficient worms for a few different staining conditions. Combine and use more 60 mm plates (or large plates) to test more conditions.
2. Wash off worms with M9 into 1.5 ml microfuge tube (if using a 60 mm plate) or 15 ml centrifuge tube (if using large plates). Wash repeatedly (5x) over 15-30 min, with the final wash step using distilled / deionized water.

**Fixation**

- If using a 1.5 ml tube, adjust total volume to 450 µl. Add 500 µl ice-cold freshly-made 2x RFB (Ruvkun-Finney buffer) + 55 µl 32% paraformaldehyde solution (Electron Microscopy Services 15714-S). [Final conc. of PFA 1.75%; original protocol uses 37% paraformaldehyde to yield 2% final concentration.]
- Snap freeze in liquid nitrogen. Tubes may now be stored at -80 C (possibly indefinitely; however, one experiment suggests it is better to proceed either immediately, or at most within a few days).
- Thaw tube(s) under warm water, or briefly in 37 C water bath. Mix by inversion, and incubate for 3.5 hr on ice with occasional mixing. [Invert tubes several times every 15-30 min.]
- Remove fix, wash 1x with TTE.

**Permeabilization**

- Add 1 ml 1% beta-mercaptoethanol in TTE (freshly-made), incubate 4 hr at 37 C with gentle mixing (e.g., Nutator or rocker).
- Wash 1x with BO_3_ (borate) buffer.
- Add 1 ml 10 mM DTT in BO_3_ Buffer (freshly-made), incubate 15 min at 37 C with gentle mixing.
- Add 1 ml 0.3% H_2_O_2_ in BO_3_ Buffer (freshly-made), incubate 15 min at RT with gentle mixing.
- Wash 2x in BO_3_ Buffer.

**Heat-Induced Antigen Retrieval (HIAR)**

- Heat in high pH solution (e.g., borate buffer at ~ pH 9), 15-30 min at 99-100 C. [Split worms into separate tubes at this step to compare with and without HIAR.]

**Blocking**

- Incubate in 200 µl antibody buffer **B,** 30 min (or longer) at RT. Remove, add 200 µl antibody buffer **A** and incubate for at least 30 min. Worms may be stored at 4 C for several months before proceeding further. [This is another point at which to split worms to test different incubation conditions.]

**Staining / Antibody incubations**

- Incubate worms in antibody buffer **A** with appropriate antibody dilution, 4 C ON.
- Wash worms 4-8 x over 1-2 hr in antibody buffer **B** at RT with gentle mixing.
- Incubate with secondary antibody at appropriate dilution in antibody buffer **A** at 4 C ON.
- Wash 8X over 2 hr with antibody buffer **B** at RT.
- Add DAPI after final wash (1 µl of 100x stock; final volume of worms in tube usually ~50-100 µl); mount on agarose pad, coverslip and view.

RECIPES – ‘Improved Finney-Ruvkun’ method*

[*NOTE: the top recipe excludes the buffer PIPES, found in other versions of the F-R protocol. This was likely an accidental omission; however, these experiments were performed using this recipe, so PIPES is not essential. A ‘corrected’ version with PIPES is also included.]

2x RFB **[No PIPES] ** – 10 mlfinal concentration

1 M KCl 1.6 ml 160 mM

5 M NaCl 80 µl 40 mM

0.5 M EGTA 0.4 ml 20 mM

1 M spermidine 0.1 ml 10 mM

methanol 5 ml 50%

H_2_O 2.82 ml

2x RFB **[with PIPES]** – 10 mlfinal conc. 

1 M KCl 1.6 ml 160 mM

5 M NaCl 80 µl 40 mM

0.5 M EGTA 0.4 ml 20 mM

0.5 M PIPES, pH 7.4 0.6 ml 30 mM

1 M spermidine 0.1 ml 10 mM

methanol 5 ml 50%

H_2_O 2.22 ml

**TTE **– 100 ml final conc.

Tris, pH 7.4 10 ml 100 mM

10% Triton X-100 10 ml 1%

EDTA 0.5 M, pH 8 200 µl 1 mM

**Borate (BO_3_) Buffer **(includes 1% Triton X-100*)

* not in the original protocol, but important to prevent loss of worms from sticking to plastic tube surfaces.

– 200 ml final conc.

0.5 M H_3_BO_3 _4 ml 0.01 M H_3_BO_3 _[Boric acid, FW 61.83]

1 M NaOH 2 ml 0.01 M NaOH

10% Triton X-100 1.5 ml 1%

**Antibody Buffer A: ** 1% BSA, 0.5% Triton X-100, 0.05% NaAzide, 1 mM EDTA in 1x PBS, pH 7.4

**Antibody Buffer B:** 0.1% BSA, 0.5% Triton X-100, 0.05% NaAzide, 1 mM EDTA in 1x PBS, pH 7.4

1x PBS, pH 7.4 [1x concentrations: 137 mM NaCl, 2.7 mM KCl, 8 mM Na_2_HPO_4_, and 2 mM KH_2_PO_4_; e.g., made from 10x PBS, Thermo-Fisher AM9625]
